# Increased Frequency of Activated Switched Memory B Cells and Its Association With the Presence of Pulmonary Fibrosis in Diffuse Cutaneous Systemic Sclerosis Patients

**DOI:** 10.3389/fimmu.2021.686483

**Published:** 2021-06-30

**Authors:** Diána Simon, Péter Balogh, Szabina Erdő-Bonyár, Katalin Böröcz, Tünde Minier, László Czirják, Tímea Berki

**Affiliations:** ^1^ Department of Immunology and Biotechnology, Clinical Center, University of Pécs Medical School, Pécs, Hungary; ^2^ Department of Rheumatology and Immunology, Clinical Center, University of Pécs Medical School, Pécs, Hungary

**Keywords:** systemic sclerosis, B cells, switched memory B cells, DN B cells, dcSSc, disease activity, natural autoantibodies, anti-topoisomerase I antibody

## Abstract

Disease-associated, high-affinity pathological autoantibody production is a well-described consequence of immune dysregulation affecting B cells in systemic sclerosis (SSc), including the distribution of B-cell subsets. We have previously shown that the increased relative frequency of CD19+CD27+IgD− switched memory B cells is associated with the severe form of SSc. This study sought to analyze memory B cell subsets using an extended range of markers for further subdivision based on CD19, IgD, CD27, CD38 and CD95 phenotype, to define relationship between the alterations of memory B cell subsets and the clinical features of SSc. Peripheral blood samples were obtained from 21 SSc patients, including 14 diffuse (dcSSc) and 7 limited (lcSSc) cutaneous SSc patients, with disease duration of 2.7 ( ± 1.6) years. After purification of CD19+ B cells, multiparametric flow cytometry was performed and the frequencies of CD19+IgD−CD27−CD38+ double negative (DN) 1, CD19+IgDloCD27+CD38+ unswitched, CD19+IgD−CD27+CD38+CD95− resting switched and CD19+IgD−CD27+CD38−CD95+ activated switched memory (ASM) B cells were determined, and correlated with clinical features of SSc. The dcSSc patients had a higher frequency of ASM B cells (p = 0.028) compared to lcSSc patients. The percentage of ASM B cells was elevated in anti-Scl-70 (anti-topoisomerase I) antibody positive patients compared to negative patients (p = 0.016). Additionally, the frequency of ASM B cells was also increased in patients with pulmonary fibrosis (p = 0.003) suggesting that patients with severe form of SSc have higher ASM B cell ratios. Furthermore, the ratio of DN1 B cells was decreased (p = 0.029), while the level of anti-citrate synthase IgG natural autoantibody was elevated (p = 0.028) in patients with active disease. Our observations on the increase of ASM B cells in dcSSc and in patients with pulmonary fibrosis may point to the association of this alteration with the severe form of the disease. Functionally the correlation of ASM B cells as effector memory-plasma cell precursors with anti-topoisomerase I antibody positivity could reflect their contribution to pathological autoantibody production, whereas the decrease of memory precursor DN B cells and the increase of anti-citrate synthase IgG autoantibody may have potential significance in the assessment of disease activity.

## Introduction

Systemic sclerosis (SSc) is a connective tissue disease with vascular damage and consequent ischemic–reperfusion injury and multiorgan fibrosis (1). Immune dysregulation is one of the hallmarks of SSc, which affects B cells, resulting in the production of scleroderma-specific autoantibodies, secretion of pro-inflammatory and pro-fibrotic cytokines. B cells can also function as antigen-presenting cells to T cells and can modify dendritic cell maturation to promote a pro-fibrotic Th2 response (2). Although elevated naive and decreased memory B cells have been reported in SSc patients compared to healthy individuals (3, 4), chronic activation, clonal expansion and SSc-specific antibody production of circulating memory B cells have been described (5), and we have previously shown that the ratio of activated memory B cells is higher in the peripheral blood of patients with diffuse cutaneous SSc (dcSSc) compared to patients with limited cutaneous SSc (lcSSc) (4). Memory B cells according to their IgD expression could be resolved into CD19+CD27+IgD− switched and CD19+CD27+IgD+ unswitched memory B cell subsets (6). We reported a relative increase in the proportion of switched memory B cells in the peripheral blood of patients with the severe form of SSc (4). SSc patients with increased percentage of CD21low B cells have recently been reported to show elevated ratio of memory B cells, encompassing higher proportions of CD27+IgM+, CD27+IgD− and CD27−IgD− memory B cells. The percentage of CD21low B cells was elevated in SSc patients with visceral vascular damage and their increase was associated with the development of new digital ulcers (7, 8).

In contrast to the above phenotyping scheme of blood B cells, in human tonsils IgD and CD38 staining is used to resolve the B cell composition into Bm1–Bm5 subsets, in which memory B cells are defined as IgD−CD38± (6, 9). As the detailed phenotyping of B cells has substantially evolved, additional markers have been suggested for the identification of further memory B cell subsets, but there is still no universal consensus on the phenotyping strategy. However, using the core markers CD19, IgD, CD38, CD27 and CD95 as an additional marker, CD19+IgD−CD27−CD38+ double negative (DN) 1, CD19+IgDloCD27+CD38+ unswitched, CD19+IgD−CD27+CD38+CD95− resting switched and CD19+IgD−CD27+CD38−CD95+ activated switched memory (ASM) B cells can be determined (10).

In humans, switched memory B cells have the propensity to differentiate into plasma cells upon reactivation (11). Based on the lower frequency of somatic hypermutation and their shared phenotype and transcriptome with switched memory B cells, DN1 cells are believed to represent early switched memory B cell precursors that have not yet acquired CD27 expression (12), while unswitched memory B cells are regarded as marginal zone (MZ) equivalent cells, also resembling B-1 cells with innate-like features (13). Several memory B cell phenotypes have been described recently, but no such data are available in SSc. Consequently, the purpose of this study was to analyze memory B cell subsets using an extended range of markers, and to search for possible associations between the alterations of memory B cell subsets, the pathological and natural autoantibody levels in peripheral blood and the clinical features of SSc.

## Materials and Methods

### Patients

Twenty-one patients with SSc (disease duration was 2.71 ( ± 1.59) years based on the date of the first non-Raynaud’s symptom) and 13 age and sex matched healthy controls (HC) were enrolled for the study. Patients were classified as dcSSc and lcSSc based on the criteria proposed by LeRoy et al. (14). None of the patients were on immunosuppressive therapy. Mean (SD) age at enrollment was 49.57 ( ± 12.4) years, mean (SD) modified Rodnan skin score was 6.4 ( ± 7.53) points, and more than half of the patients had ILD. We assessed pulmonary arterial hypertension, pulmonary fibrosis, scleroderma renal crisis and skin involvement according to our standard protocol (15). Disease activity was recorded according to the physician’s opinion based on internal organ, skin, and musculoskeletal manifestations. The detailed characteristics of the patients are shown in **Table 1**. All participants gave a written informed consent to the study, after approval by the Research Ethics Committee of the Hungarian Medical Research Council (ETT-TUKEB) (84-256/2008-1018EKU).

**Table 1:** Patients’ characteristics.

	SSc (*n* = 21)	dcSSc (n = 14)	lcSSc (n = 7)
**General data**			
Age (years), mean (SD)	49.57 (12.4)	50 (13.8)	48.71 (9.96)
Gender (female), *n* (%)	17/21 (80.95%)	10/14 (71.43%)	7/7 (100%)
Disease duration^1^ (years), mean (SD)	2.71 (1.59)	2.86 (1.7)	2.43 (1.4)
Active disease^2^, n (%)	10/21 (47.62%)	7/14 (50%)	3/7 (42.86%)
EScSG-AI ≥3^3^, n (%)	6/21 (28.57%)	5/14 (35.71%)	1/7 (14.29%)
**Organ involvement**			
MRSS mean (SD)	6.4 (7.53)	8.43 (8.21)	1.67 (1.37)
Lung fibrosis^4^, *n* (%)	11/21 (52.38%)	9/14 (64.29%)	2/7 (28.57%)
Pulmonary arterial hypertension (PAH)^5^, *n* (%)	1/21 (4.76%)	1/14 (7.14%)	0/7 (0%)
Renal involvement^6^, *n* (%)	1/21 (4.76%)	1/14 (7.14%)	0/7 (0%)
**Antibodies**			
Anti-Scl-70+, *n* (%)	6/21 (28.57%)	5/14 (35.71%)	1/7 (14.29%)
Anti-RNA-polymerase III+, *n* (%)	2/21 (9.52%)	2/14 (14.29%)	0/7 (0%)
anti-centromere+, *n* (%)	8/21 (38.1%)	3/14 (21.43%)	5/7 (71.43%)

^1^Onset of the disease was defined as the date of the first non-Raynaud’s symptom.

^2^According to physician’s opinion based on organ manifestations.

^3^Active disease based on EScSG-AI (16).

^4^Pulmonary fibrosis was characterized by detection of fibrosis with high resolution CT and/or decreased forced vital capacity (FVC <80%).

^5^Right ventricle pressure ≥25 Hgmm measured by right heart catheterization.

^6^Scleroderma renal crisis was recorded as kidney involvement.

### Peripheral Blood Mononuclear Cell Isolation and B Cell Separation

Peripheral blood mononuclear cells (PBMCs) were isolated by Ficoll–Paque Plus (GE Healthcare, Chicago, IL, USA) density gradient centrifugation of peripheral blood samples. EasySep™ Human CD19 Positive Selection Kit (Stemcell Technologies, Vancouver, BC, Canada) was used for positive selection of CD19+ cells according to the manufacturer’s instructions achieving >95% purity.

### Phenotyping of Memory B Cell Subsets

After checking the purity of the separated B cells using anti-human CD20-PE (L27, Becton Dickinson, Franklin Lakes, NJ, USA) multiparametric flow cytometry was performed on the highly purified CD19+ B cells to investigate memory B cell subsets. The phenotyping analysis of memory B cells was conducted using the combination of anti-human IgD-PE (IA6-2, Becton Dickinson, Franklin Lakes, NJ, USA), anti-human CD27-PE/Cy5 (O323, Thermo Fisher Scientific, Waltham, MA, USA), anti-human CD38-APC (HIT2, Thermo Fisher Scientific, Waltham, MA, USA) and anti-human CD95-FITC (DX2, Thermo Fisher Scientific, Waltham, MA, USA) following the manufacturer’s protocols. Briefly, CD19+ B cells were incubated with the appropriate antibodies for 30 min on ice, washed twice in phosphate buffered saline and fixed with 1% paraformaldehyde. Fluorescence of the labelled cells was recorded using a FACS Calibur flow cytometer (Becton Dickinson, Franklin Lakes, NJ, USA) and analyzed using FCS Express 6 software (De Novo Software, Pasadena, CA, USA).

### Measurement of Pathological and Natural Autoantibodies

The presence of anti-topoisomerase I (topo I), anti-centromere and anti-RNA polymerase III antibodies in the serum sample of patients were measured using commercial ELISA (ORG 546 and 633 Orgentec Diagnostika, Mainz, Germany) and immunoblot kits (Euroline Systemic Sclerosis Profile, Euroimmun, Lübeck, Germany) according to the manufacturer’s protocol. The levels of anti-citrate synthase (anti-CS) IgG autoantibodies were determined with an in-house ELISA, as previously described (17); ninety-six-well polystyrene plates (Nunc, Roskilde, Denmark) were coated with 100 µl of 5 µg/ml citrate synthase from porcine heart (Sigma, St. Louis, MO, USA) in 0.1 M bicarbonate buffer, pH 9.6 at 4–8°C overnight. Following the saturation of non-specific binding sites with 0.5% gelatin (Sigma) in phosphate buffered saline (PBS) (pH 7–3), serum samples were incubated in duplicate at 1:100 dilution in washing buffer (PBS, 0.05% Tween 20) for 1 h at room temperature. Finally, after extensive washing the plate was incubated with horseradish peroxidase (HRP)-conjugated anti-human IgG-specific secondary antibody (Dako, Glostrup, Denmark) for 1 h at room temperature. The reaction was developed with TMB and measured at 450 nm, using an iEMS MF microphotometer (Thermo Labsystem, Beverly MA, USA). Five-point dilution series of our in-house anti-CS standard was used for quantification.

### Statistical Analysis

Statistical evaluation was performed using SPSS version 25.0 statistics package (IBM, Armonk, NY, USA). Mann–Whitney U tests were used and p values <0.05 were considered significant.

## Results

### Changes in the Frequency of Memory B Cell Subsets in SSc

**Figure 1:**
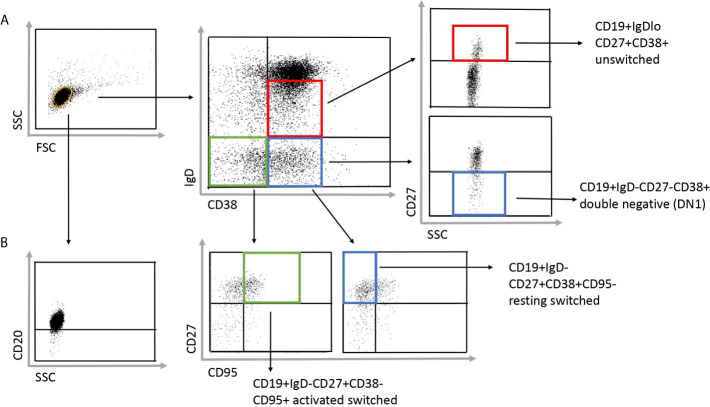
Gating strategy to identify the investigated four subgroups of memory B cells in SSc patients. **(A)** The CD19+ purified B cells were stained with IgD, CD38, CD27 and CD95 defining the following subsets: CD19+IgDloCD27+CD38+ unswitched, CD19+IgD−CD27−CD38+ double negative (DN) 1, CD19+IgD−CD27+CD38+CD95− resting switched and CD19+IgD−CD27+CD38−CD95+ activated switched memory B cells. **(B)** To check the purity of the separated B cell, cells were stained with anti-CD20 antibody after separation.

**Figure 2:**
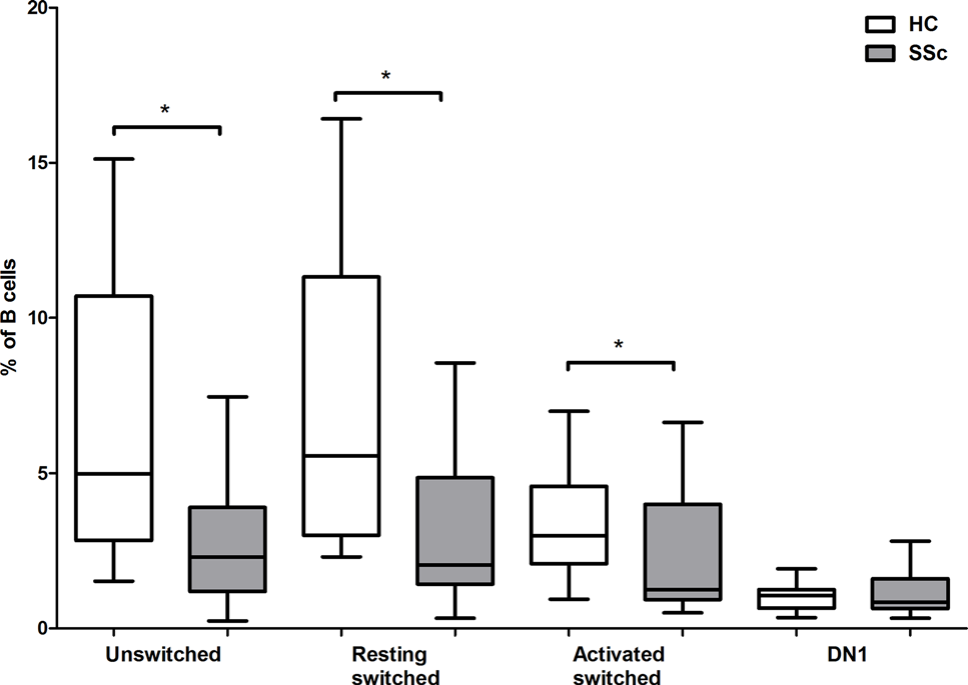
Changes in the percentage of memory B cell subsets in SSc compared to HC. The distribution of CD19+IgDloCD27+CD38+ unswitched, CD19+IgD−CD27−CD38+ double negative (DN) 1, CD19+IgD−CD27+CD38+CD95− resting switched and CD19+IgD−CD27+CD38−CD95+ activated switched memory B cells are shown in HCs (n = 13) and in SSc patients (n = 21). The boxes show interquartile ranges (IQR), the horizontal lines represent medians and the whiskers indicate the lowest and highest values. *p <0.05.

To investigate the distribution of memory B cell subsets in the peripheral blood of SSc patients and HCs the frequencies of CD19+IgD−CD27−CD38+ double negative (DN) 1, CD19+IgDloCD27+CD38+ unswitched, CD19+IgD−CD27+CD38+CD95− resting switched and CD19+IgD−CD27+CD38−CD95+ activated switched memory (ASM) B cells were determined (**Figure 1A**) in highly enriched B cells (**Figure 1B**). First, we analyzed the alterations of peripheral blood memory B cell subsets in SSc compared to HC, and found that the percentage of unswitched, resting switched and ASM B cells was significantly lower in SSc than in HC (p = 0.008, p = 0.002 and p = 0.044 respectively), while there was no significant difference in the proportion of DN 1 cells between SSc and HC samples (**Figure 2**).

### The Distribution of Memory B Cell Subsets Is Altered in dcSSc and Correlates With Anti-Topoisomerase I Autoantibody Positivity

**Figure 3:**
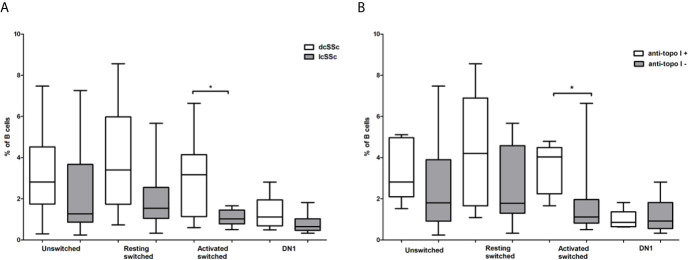
Differences in the frequency of memory B cell subsets in defined subgroups of SSc. CD19+IgDloCD27+CD38+ unswitched, CD19+IgD−CD27−CD38+ double negative (DN) 1, CD19+IgD−CD27+CD38+CD95- resting switched and CD19+IgD−CD27+CD38−CD95+ activated switched memory B cells were analyzed **(A)** in dcSSc (n = 14) and lcSSc (n = 7) patients and **(B)** in anti-topoisomerase I (topo I) positive (n = 6) and anti-topo I negative (n = 15) SSc patients. The boxes show interquartile ranges (IQR), the horizontal lines represent medians and the whiskers indicate the lowest and highest values. *p <0.05.

We looked for relationship between the alterations of memory B cell subsets and the serological features of SSc. First, we compared the proportion of memory B cell subsets in dcSSc and lcSSc. We found that the only significant difference was in the percentage of ASM B cells, which was significantly elevated in dcSSc (median: 3.16, interquartile range (IQR): 1.13–4.14) compared to lcSSc (median: 1.02, IQR: 0.78–1.45) (p = 0.028) (**Figure 3A**). Since anti-Scl-70 (anti-topo I) antibodies are considered to be associated with dcSSc (18) we also compared the proportion of memory B cell subsets in anti-topo I positive and negative patients, and found that the percentage of ASM B cells was significantly elevated in anti-topo I positive (median: 4.03, IQR: 2.24–4.49) compared to anti-topo I negative (median: 1.11, IQR: 0.82–1.97) SSc patients (p = 0.016) (**Figure 3B**). Male patients with SSc are reported to have a more severe disease (19), but even when only female patients were included in the analysis, the significant differences in association with dcSSc could still be observed (data not shown).

### Increased Activated Switched Memory B Cells in SSc Patients With Interstitial Lung Disease

**Figure 4:**
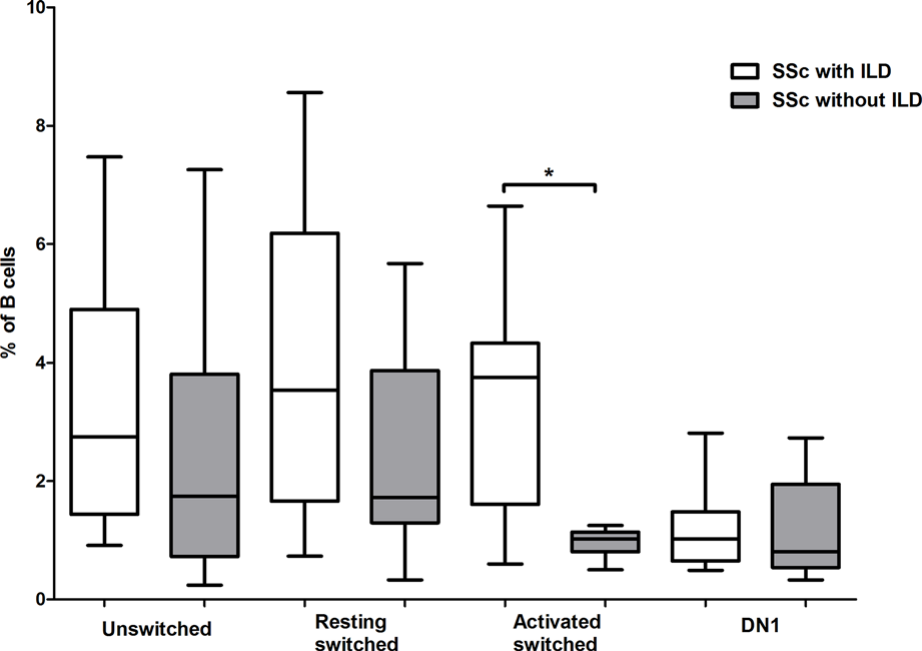
Alterations in the percentage of memory B cell subsets in patients with interstitial lung disease (ILD). The distribution of CD19+IgDloCD27+CD38+ unswitched, CD19+IgD−CD27−CD38+ double negative (DN) 1, CD19+IgD−CD27+CD38+CD95− resting switched and CD19+IgD−CD27+CD38−CD95+ activated switched memory B cells are shown in SSc patients with ILD (n = 11) and without ILD (n = 10). The boxes show interquartile ranges (IQR), the horizontal lines represent medians and the whiskers indicate the lowest and highest values. *p <0.05.

Interstitial lung disease (ILD) is the most common cause of death among patients with SSc, and it is more common in dcSSc, or in patients positive for anti-Scl-70 antibodies (20), thus we analyzed the alteration of memory B cell subsets in ILD. ILD was defined by detection of fibrosis with high resolution CT and/or decreased forced vital capacity (FVC <80%). According to our results, the pattern of differences in the proportion of memory B cell subsets between patients with and without ILD are similar to the pattern found when comparing dcSSc to lcSSc, and anti-topo I positive to anti-topo I antibody negative patients. Significant difference was only found in the percentage of ASM B cells, which was elevated in patients with ILD (p = 0.003) (**Figure 4**). All of these patients with ILD had pulmonary fibrosis on HRCT evaluated by radiologists, and the average ± SD values of FVC and the diffusing capacity of the lungs for carbon monoxide (DLCO) of these patients were 92 ± 12.86 and 56.82 ± 19.74 respectively.

### DN1 B Cells Are Decreased in Patients With Active Disease

**Figure 5:**
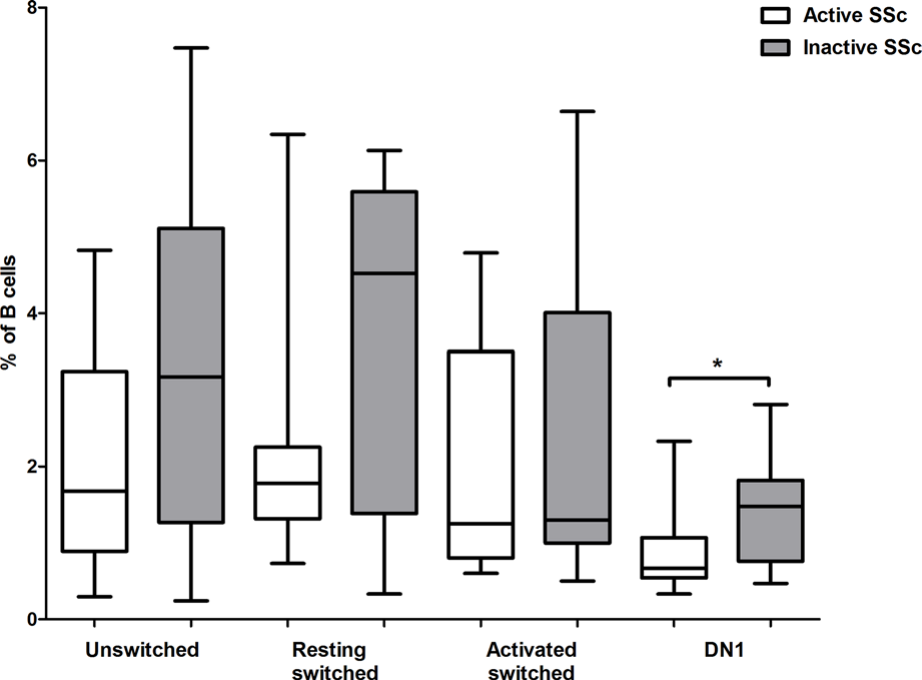
Changes in the proportion of memory B cell subsets in active disease. The percentages of CD19+IgDloCD27+CD38+ unswitched, CD19+IgD−CD27−CD38+ double negative (DN) 1, CD19+IgD−CD27+CD38+CD95− resting switched and CD19+IgD−CD27+CD38−CD95+ activated switched memory B cells were analyzed in active (n = 10) and inactive (n = 11) SSc patients. The boxes show interquartile ranges (IQR), the horizontal lines represent medians and the whiskers indicate the lowest and highest values. *p <0.05.

Assessment of disease activity in SSc is challenging due to the heterogeneity of clinical manifestations, and the definition of the concept of disease activity in SSc is inconsistent in the literature (21). We used the EUSTAR Activity Index (EScSG-AI) to assess disease activity in the enrolled SSc patients (16). We also verified the disease activity according to the physician’s opinion based on internal organ involvement and skin and musculoskeletal manifestations. We analyzed the distribution of memory B cell subsets in patients with active and inactive disease, and found no significant differences in the percentage of the investigated memory B cell subsets between patients with active and inactive disease according to EScSG-AI (data not shown). However, the percentage of DN1 B cells was significantly lower in patients with active disease based on the opinion of the physician (p = 0.029) (**Figure 5**).

### The Level of Anti-Citrate Synthase IgG Natural Autoantibody Is Elevated in Patients With Active Disease

**Figure 6:**
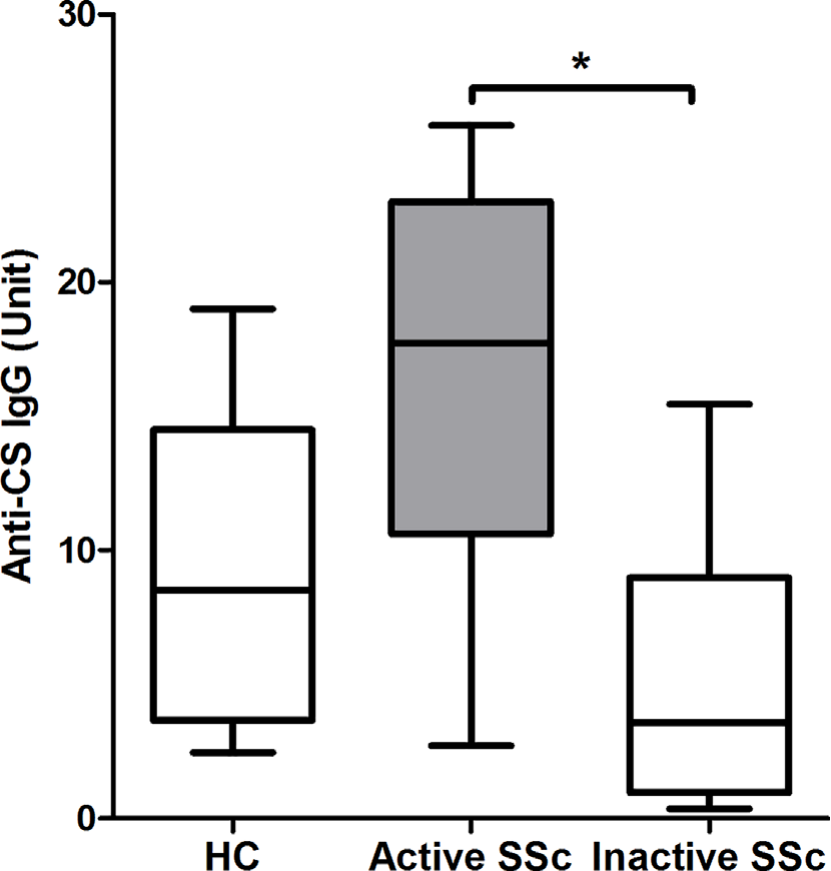
Relationship between disease activity and anti-citrate synthase (CS) IgG antibody titers in SSc patients (n = 14) and HCs (n = 13). The boxes show interquartile ranges (IQR), the horizontal lines represent medians and the whiskers indicate the lowest and highest values. *p <0.05.

Levels of natural autoantibodies with IgG isotype were shown to fluctuate over time (22) and their amount showed association with subgroups of systemic lupus erythematosus (SLE) patients (23). Therefore, we looked for differences in the level of anti-CS IgG antibodies representing natural autoantibody (15, 17) in defined SSc subgroups. First, we measured the level of anti-CS IgG antibodies in serum samples of HCs and patients with SSc and found no significant difference (HC: median = 8.53, range = 16.54; SSc: median = 7.89, range = 25.49; p = 0.877). We also compared the amount of anti-CS IgG antibodies in subgroups of SSc patients, such as dcSSc or lcSSc, anti-topo I+ or anti-topo I-cases, and we also evaluated the possible associations between the amount of anti-CS IgG antibodies in the serum of patients with different organ manifestations without significant results (data not shown). However, the anti-CS IgG level was significantly higher in patients with active disease than in patients with inactive disease (p = 0.028) (**Figure 6**). Additionally, comparison of anti-CS IgG level in active, inactive SSc patients and HCs showed a higher tendency in active SSc than in HC (p = 0.083) (**Figure 6**).

## Discussion

Dysregulated B cells are early contributors in the pathogenesis of SSc (24). Chronic activation, clonal expansion, and antibody production of circulating memory B cells have been reported (5) but studies on memory B cell subsets in SSc are scarce. CD27−IgD− DN B cells have been reported to be elevated in SSc compared to HCs (25). The DN B cells are also increased in SLE patients and are associated with higher disease activity and disease-specific autoantibodies (26). The DN cell expansion reflects a subset of DN2 cells in SLE (12), which are believed to represent extrafollicular antibody-secreting cell precursors (10). While only the minority of DN cells were DN2 cells in HCs, their fraction was greater in SLE (12). The proportion of DN2 cells was also found to be elevated in SSc (12), consequently the increased frequency of DN cells in dcSSc compared to lcSSc in our previous study (4) could be explained by the expansion of DN2 cells. In agreement with this, we found no significant differences in the frequency of DN1 cells either between HC and SSc, or dcSSc and lcSSc patients; however, the percentage of DN1 cells was lower in patients with active than in patients with inactive disease. ASM B cells are described as effector memory-plasma cell precursors (10) and although we found that their frequency is lower in SSc than in HC, we demonstrated their elevated proportion in dcSSc patients, in patients with anti-topo I antibody positivity and pulmonary fibrosis, thus they may represent a potential biomarker for the severe form of SSc. Also, anti-topo I antibody positivity was reported to be associated with HLA DPB1*1301 haplotype (27, 28), therefore HLA DPB1*1301 haplotype might indicate a predisposition to increased percentage of ASM B cells in severe form of SSc.

Natural autoantibodies are present in the serum of both healthy individuals and patients with systemic autoimmune diseases. They recognize evolutionally conserved self-structures, most of them belong to the IgM isotype, and are polyreactive. Natural autoantibodies have been described to have several functions in the immune system, including clearance of apoptotic debris, acceleration of primary immune responses, suppression of autoimmune and inflammatory responses, regulation of B cell responses and B cell development (29). Natural autoantibodies of IgG isotype have also been described (30, 31), their presence in human sera is thought to represent a breakdown in central tolerance (30), and the intensity of their reactivity to self-antigens is higher in patients than in healthy individuals (32). The structural and functional conservation of mitochondrial components makes citrate synthase (CS) a candidate antigen for detailed analysis of physiological autoreactive immune response and their changes under pathological autoimmune conditions to molecules representing an interesting transition from prokaryotic foreign to essential self-antigens (17). In our previous studies, we detected natural anti-CS antibodies in healthy individuals and in patients with systemic autoimmune diseases (17, 23). B-1 cells produce natural IgM from unmutated or minimally mutated V(D)J genes, but they can undergo somatic hypermutation and class switching and produce natural IgG or IgA autoantibodies with higher affinity (33), particularly in autoimmune diseases (34). In addition to B-1 cells, natural antibodies can be produced by MZ B cells (13). As unswitched memory B cells in human peripheral blood represent MZ derived B cells (35), we correlated the level of anti-CS IgG with the percentage of unswitched memory B cells, but without significant result, consequently both B-1 and unswitched memory B cells could participate in the anti-CS Ig antibody production in SSc. In our previous study, we found a positive association between the level of anti-CS and anti-dsDNA IgG levels in SLE (23). Anti-dsDNA antibodies are associated with active SLE (36) and here we found that the anti-CS Ig antibodies are elevated in active SSc compared to inactive SSc and HC. Under pathological conditions a compensatory increase in IgG antibodies with anti-idiotypic activity can occur (37); however, at a certain point even the increased amount of anti-idiotypic IgG may not be able to ameliorate disease activity mediated by pathogenic IgG autoantibodies. Intravenous immunoglobulin (IVIG) seems to be beneficial in SSc (38), and IVIG is rich in natural antibodies (39), thus it may exert its favorable effects by supporting the natural antibody pool of patients as given the altered physiology in autoimmune patients, it is plausible that these natural autoantibodies are needed at higher amounts (29).

Assessment of disease activity in SSc is still a challenge, and EScSG-AI showed only moderate correlation with the score given by a physician when assessing disease activity globally in a large Canadian cohort (40) and the index was criticized for not considering, for example, renal involvement. We found no association between disease activity and the investigated memory B cell subsets using EScSG-AI to assess disease activity. However, the percentage of DN1 B cells was lower in patients with active disease when disease activity was evaluated according to the physicians’ opinion based on internal organ, skin, and musculoskeletal manifestations. There is still a need for a valid composite activity index for the adequate assessment of disease activity in every potentially involved organ in SSc, and according to our results, the determination of DN1 B cell ratio and the measurement of natural anti-CS IG antibody level could supplement the currently available indexes.

## Data Availability Statement

The original contributions presented in the study are included in the article/supplementary material. Further inquiries can be directed to the corresponding author.

## Ethics Statement

The studies involving human participants were reviewed and approved by the Research Ethics Committee of the Hungarian Medical Research Council (ETT-TUKEB). The patients/participants provided their written informed consent to participate in this study.

## Author Contributions

DS and PB performed the B cell separation and flow cytometry experiments. SE-B and KB carried out the autoantibody measurements. TM and LC contributed the clinical data. DS, PB and SE-B analyzed the data. DS planned and designed experiments. TB and PB provided critical oversight of experiments. DS and SE-B wrote the manuscript. PB and BT reviewed the manuscript. All authors contributed to the article and approved the submitted version.

## Funding

This research was funded by GINOP-232-15-2016-00050 and EFOP 361-16-2016-00004 and by TKP2020-IKA-08 supported by the National Research, Development and Innovation Fund of Hungary under the 2020-4.1.1-TKP2020 funding scheme.

## Conflict of Interest

The authors declare that the research was conducted in the absence of any commercial or financial relationships that could be construed as a potential conflict of interest.
